# Multidimensional OMICs reveal ARID1A orchestrated control of DNA damage, splicing, and cell cycle in normal‐like and malignant urothelial cells

**DOI:** 10.1002/1878-0261.70019

**Published:** 2025-04-01

**Authors:** Rebecca M. Schlösser, Florian Krumbach, Eyleen Corrales, Geoffroy Andrieux, Christian Preisinger, Franziska Liss, Alexandra Golzmann, Melanie Boerries, Kerstin Becker, Ruth Knüchel, Stefan Garczyk, Bernhard Lüscher

**Affiliations:** ^1^ Institute of Pathology, RWTH Aachen University Hospital Aachen Germany; ^2^ Institute of Medical Bioinformatics and Systems Medicine, Medical Center – University of Freiburg, Faculty of Medicine University of Freiburg Germany; ^3^ Proteomics Facility, Interdisciplinary Centre for Clinical Research (IZKF), Medical School RWTH Aachen University Aachen Germany; ^4^ Institute of Biochemistry and Molecular Biology, Faculty of Medicine RWTH Aachen University Aachen Germany; ^5^ German Cancer Consortium (DKTK), Partner Site Freiburg, a Partnership between DKFZ and Medical Center – University of Freiburg Germany; ^6^ Cologne Center for Genomics (CCG), Medical Faculty University of Cologne Germany; ^7^ Center for Integrated Oncology Aachen Bonn Cologne Duesseldorf (CIO ABCD) Aachen Germany; ^8^ Present address: Theodor Boveri Institute and Comprehensive Cancer Center Mainfranken Biocenter University of Wuerzburg Germany

**Keywords:** ARID1A, ATAC‐Seq, bladder cancer, DNA‐damage checkpoints, RNA‐Seq, SWI/SNF

## Abstract

Epigenetic regulators, such as the SWI/SNF complex, with important roles in tissue development and homeostasis, are frequently mutated in cancer. ARID1A, a subunit of the SWI/SNF complex, is mutated in approximately 20% of all bladder tumors; however, the consequences of this remain poorly understood. Finding truncations to be the most common mutation, we generated loss‐ and gain‐of‐function models to conduct RNA‐Seq, interactome analyses, Omni‐ATAC‐Seq, and functional studies to characterize ARID1A‐affected pathways potentially suitable for the treatment of ARID1A‐deficient bladder cancers. We observed decreased cell proliferation and deregulation of stress‐regulated pathways, including DNA repair, in ARID1A‐deficient cells. Furthermore, ARID1A was linked to alternative splicing and translational regulation on RNA and interactome levels. ARID1A deficiency drastically reduced the accessibility of chromatin, especially around introns and distal enhancers, in a functional enrichment analysis. Less accessible chromatin areas were mapped to pathways such as cell proliferation and DNA damage response. Indeed, the G2/M checkpoint appeared impaired after DNA damage in ARID1A‐deficient cells. Together, our data highlight the broad impact of ARID1A loss and the possibility of targeting proliferative and DNA repair pathways for treatment.

## Graphical abstract

Loss of the frequently mutated chromatin remodeler ARID1A, a subunit of the SWI/SNF cBAF complex, results in less open chromatin, alternative splicing, and the failure to stop cells from progressing through the cell cycle after DNA damage in bladder (cancer) cells. Created in BioRender.

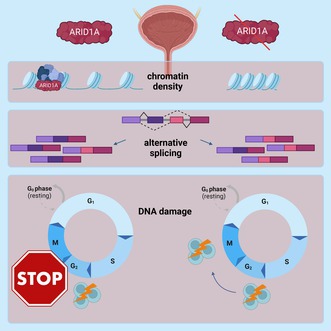

**Abbreviations**
ARID1A: AT‐rich interactive domain‐containing protein 1A
ARID1B: AT‐rich interactive domain‐containing protein 1B
ARM repeats: Armadillo repeats
ATR: ataxia telangiectasia and RAD3‐related protein
cBAF: canonical BRG1/BRM‐associated factor complex
CFA: colony formation assay
CHK1: checkpoint kinase 1
Co‐IP: co‐immunoprecipitation
CTCF: CCCTC‐binding factor
EMT: epithelial‐mesenchymal transition
GEO: Gene Expression Omnibus
GO: gene ontology
GSEA: gene set enrichment analysis
IFN: interferon
IR: ionizing radiation
ISGs: interferon‐stimulated genes
KD: knockdown
KO: knockout
MSK: Memorial Sloan Kettering
ncBAF: non‐canonical BAF
Omni‐ATAC‐Seq: Omni assay for transposase‐accessible chromatin by sequencing
PAM: Protospacer Adjacent Motif
PBAF: polybromo‐associated BAF complex
PBS: phosphate‐buffered saline
PRC2: Polycomb Repressive Complex 2
PRPF40: pre‐mRNA‐processing factor 40
RNAPII: RNA polymerase II
RNA‐Seq: RNA sequencing
snRNP: small nuclear ribonucleoprotein
SWI/SNF complex: SWItch/Sucrose Non‐Fermentable complex
TBS‐T: tris‐buffered saline (with tween)
TSS: transcription start sites

## 1 Introduction

Genes encoding chromatin‐modifying proteins are frequently mutated in cancer, functioning as tumor suppressors or oncoproteins, respectively [1]. Analyzing 44 exome sequencing studies from different tumors, Kadoch *et al*. found subunits of chromatin‐modifying mammalian SWI/SNF complexes to be mutated in approximately 20% of all cases [2]. The SWI/SNF complex was first identified in yeast in 1984 and demonstrated to be required for mating‐type switching [3, 4]. Highly conserved in eukaryotes, SWI/SNF complexes utilize ATP hydrolysis to modify chromatin by mobilizing nucleosomes, resulting in their repositioning (sliding) or ejection [5, 6].

SWI/SNF complexes can be subdivided into the canonical BRG1/BRM‐associated factor complex (cBAF), the polybromo‐associated BAF complex (PBAF), and the non‐canonical BAF complex (ncBAF) depending on their subunit composition [7, 8, 9]. Currently, 29 genes are known to encode proteins of these three complexes that possess at least 10 subunits. Some subunits are part of a common core complex, whereas others are complex‐specific [8, 9, 10]. The mutually exclusive proteins AT‐rich interacting domain‐containing protein 1A and 1B (ARID1A/BAF250a and ARID1B/BAF250b, respectively) are subunits of cBAF complexes. Importantly, *ARID1A* encodes the most frequently mutated SWI/SNF subunit [2, 11, 12]. While somatic mutations occur in many cancers, heterozygous germline mutations of *ARID1A* or *ARID1B* have been linked to the neurodevelopmental disorder Coffin‐Siris syndrome [2, 13]. Interestingly, *ARID1A* mutations are especially prevalent in bladder cancer with frequencies of 15–25% [2, 14, 15].

**Fig. 1:**
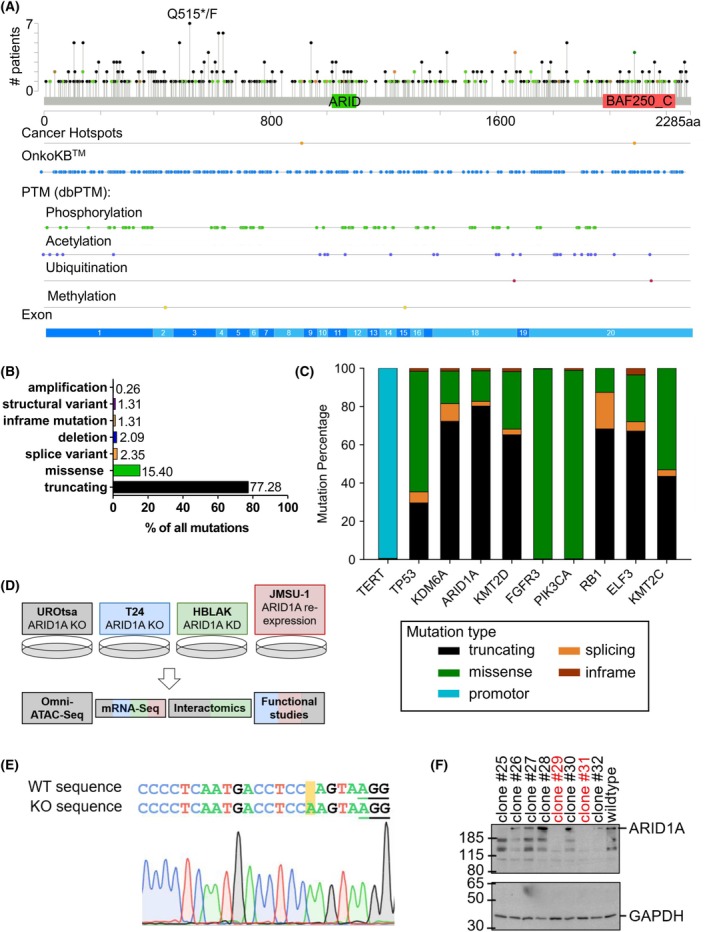
Analysis of bladder tumor‐associated ARID1A mutations reveals high prevalence of truncations. (A–C) Data were derived from the bladder cancer data of the MSK panel 2022 (*n* = 619 including 131 duplicate mutations in patients with multiple samples) [23]. (A) Lollipop plot of ARID1A mutations with Cancer Hotspots, OncoKB, and post‐translational modification annotation downloaded from cbioportal [24, 25, 26]; (B) ARID1A mutation types; (C) Mutation types of the top 10 mutated genes; (D) Study design summarizing the generation of the different cell line models developed here and the subsequent analysis performed (color coded); (E) Representative Sanger sequencing result of UROtsa ARID1A KO #5, the PAM (Protospacer Adjacent Motif) is underlined and the difference highlighted in yellow; (F) Western blot imaging of potential UROtsa ARID1A knockout cells and wildtype control; clones indicated in red are knockout clones, which were selected for further studies.

Two domains of ARID1A have been previously described (Fig. 1A). The first is the conserved ARID domain, which is responsible for DNA binding in a sequence unspecific manner [12, 16, 17, 18]. Chandler *et al*. found that substituting valine at position 1068 for glycine (V1068G) in the murine ARID domain results in severe impairment of DNA binding, although ARID1A is still incorporated into the cBAF complex [19]. Similar findings were made when the equivalent mutant, V1067G, was introduced in human ARID1A [20]. The second domain is referred to as BAF250_C (or DUF3518), a C‐terminal region with conserved surface‐exposed residues, which has been predicted to fold as Armadillo (ARM) repeats and hypothesized to possess a role in protein–protein interactions with, for example, BAF60B, BRG1, and BAF53B [20, 21]. *ARID1A* is strongly affected by mutations, and even C‐terminal mutations can lead to either protein loss or a substantially lower expression [22].

In different studies, SWI/SNF complexes have been found located at promoters, transcription start sites (TSS), and enhancers. These findings are consistent with SWI/SNF complexes being associated with loci that are occupied by CCCTC‐binding factor (CTCF) and RNAPII. The positioning at the different sites has been suggested to depend on the composition of the SWI/SNF complexes [27, 28, 29]. PBAF complexes, for example, are mostly located at promoters, ncBAF at CTCF sites and promoter regions, and cBAF complexes at enhancers [30]. The binding of cBAF complexes to chromatin is promoted by RNAPII and transcription factors [31].

ARID1A influences DNA damage repair in different ways [12, 32]. It has been shown that it facilitates R‐loop resolution, which then allows DNA repair [32]. Furthermore, enrichment of ARID1A was observed at double‐strand breaks, at least in part dependent on ATR (ataxia telangiectasia and RAD3‐related protein). Although ATR primarily recognizes single‐strand breaks, it can facilitate double‐strand break end resection [33, 34]. ATR and its downstream effector CHK1 (checkpoint kinase 1) inhibit cell cycle progression and prevent damaged cells from entering mitosis. Therefore, it has been argued that ARID1A‐deficient cancers are sensitive to ATR inhibitors, which, in turn, promote apoptosis [35, 36]. Indeed, a phase I and a phase II clinical trial have tested the ATR inhibitor ceralasertib (AZD6738) in patients with ARID1A loss in different cancers and show durable anti‐tumor activity [37, 38].

While ARID1A supports RNA polymerase II ejection after DNA damage of transcribed regions, ARID1A deficiency has been reported to result in increased transcription of genes encoding pro‐proliferative factors [39]. Surprisingly, the loss of ARID1A indirectly inhibits the expression of the eukaryotic elongation factor 2, resulting in translational inhibition. This latter effect, which is tumor suppressive, can be overcome by increasing the translational speed through mutating genes of other tumor‐associated key factors such as TP53, PTEN, or APC, which promote cell proliferation [39]. These important findings were evaluated in preclinical studies, revealing that ARID1A‐deficient tumors are particularly sensitive to protein synthesis inhibitors [39].

Besides modulating transcription, ARID1A has also been found to be associated with PRPF40 (pre‐mRNA‐processing factor 40), which is part of the U2 small nuclear ribonucleoprotein (snRNP) complex, a key component of the spliceosome [40]. This suggests that ARID1A is, besides controlling chromatin and transcription, also involved in splicing.

In addition to the activities described above, which affect tumor cell‐intrinsic functions, ARID1A deficiency has been reported to influence the interaction of tumor cells with immune cells. An increase in tumor‐infiltrating lymphocytes was noted in a study of ovarian cancer in syngeneic mice [21, 41], while inhibited CD8+ T cell infiltration was seen in triple‐negative breast cancer patients with low ARID1A expression [42]. These important differences might be the result of the distinct cell systems analyzed.

Due to the suggested antagonistic activities on various cellular pathways, ARID1A may act as a tumor suppressor or oncoprotein dependent on the cellular context. Here, we compared the effects of ARID1A depletion and re‐expression in normal‐like and tumor‐like urothelial bladder cell models to gain further insight regarding the consequences of *ARID1A* inactivation and to identify pathways that might serve as possible targets for treatment. As *ARID1A* mutations can already be observed in early bladder cancer lesions, it is important to address the consequences of ARID1A deregulation as it might help to understand how early lesions progress towards muscle‐invasive disease with poor prognosis [15, 23, 43].

## 2 Materials and methods

All oligo sequences (DNA, siRNA, gRNA, primers) can be found in Table S1.

### 2.1 Cell cultures

All human cell lines were tested regularly for mycoplasma contamination and were verified with the Multiplex human Cell line Authentication Test (MCA) (Multiplexion). UROtsa (RRID:CVCL_0571 [44, 45, 46], normal‐like from a primary culture, immortalized by SV40 large T‐antigen [47]) and T24 (RRID:CVCL_0554 [44, 45, 46], malignant [48]) cells were cultured in RPMI1640 (Gibco, Germany) supplemented with 10% FCS (PAN‐Biotech, Aidenbach, Germany) and 1% Penicillin–Streptomycin–Glutamine (Thermo Fisher Scientific, Germany). UROtsa are described as non‐tumorigenic due to their lack of growth in soft agar and their inability to form tumors in nude mice [49]. For JMSU‐1 (RRID:CVCL_2081 [44, 45, 46], metastatic [50]), 3 μg·mL^−1^ blasticidine (Invitrogen, Germany) was added. HBLAK (RRID:CVCL_JQ59 [44, 45, 46], normal‐like from a primary culture, spontaneously immortalized [51]) were cultured in CnT Prime (CellnTech, Bern, Switzerland). All cells were passaged twice a week at a confluence of 70–90% and grown at 37 °C with 5% CO_2_. ARID1A knockout, knockdown, and re‐expressing cells were generated in this study.

### 2.2 Transfection of cells

1 × 10^5^ T24 cells were transfected with 3 μL FuGene (Promega, Walldorf, Germany) and 1 μg PX459 ARID1A KO in RPMI1640 (Gibco) in 6‐well plates. Puromycin selection (Thermo Fisher Scientific; 0.5 μg·mL^−1^) was started 72 h post‐transfection, and single‐cell clones were selected.

70–90% confluent UROtsa cells were transfected using the Neon Transfection System (Invitrogen). 1 × 10^7^ cells·mL^−1^ in buffer R were gently mixed with 5 μg PX458 ARID1A KO and 5 μg pUC19 to enhance transfection efficiency (as reported in [52]). Neon Tubes were filled with 3 mL buffer E2 and cells were electroporated in 100 μL tips with 1400 V for 20 ms for 2 pulses. After 24 h of growth in antibiotic‐free RPMI1640 (Gibco), cells were sorted for GFP positivity with a BD FACS Aria II (BD, Heidelberg, Germany, 3‐laser, 9‐color (2‐5‐2)), and single‐cell clones were selected.

HBLAK cells were seeded at 1.2 × 10^5^ cells in 6‐well plates. On the following day, 24 μL ARID1A siRNA (5 mm) was mixed with 70 μL CnT Prime (CellnTech, Bern, Switzerland) and then 6 μL HiPerfect (Qiagen, Hilden, Germany). The mix was added to the cells after a brief vortexing and a 10‐min incubation at room temperature, resulting in a final siRNA concentration of 50 nm. Cells were grown for another 72 h for RNA‐Seq.

80% confluent JMSU‐1 cells in RPMI1640 (Gibco, Germany) supplemented with 10% FCS were transfected with pcDNA6‐ARID1A (addgene) using FuGene HD (Promega). Blasticidine (Invitrogen) selection was performed.

### 2.3 Western blotting

Western blotting was performed according to [53] with PBS instead of TBS. All antibodies were diluted in TBS‐T with 1% milk powder. ARID1A (D2A8U, 1:1000, 12 354; Cell Signaling, Leiden, The Netherlands) and GAPDH (14C10, 1:50000, 2118, Cell Signaling) or Beta‐actin (1:1000, A5441; Sigma‐Aldrich, Taufkirchen, Germany) were used as primary antibodies. Acquisition was done on film or visualized by an iBright™ Imager (Thermo Fisher Scientific).

### 2.4 Sanger sequencing

DNA was harvested from confluent plates, and DNA was isolated using the QIAmp DNA Mini Kit (Qiagen). PCR amplification was performed with GoTaq (Promega). Primers and conditions are summarized in Table S1. DNA was purified from nucleotides with ExoCIP (NEB, Frankfurt am Main, Germany) and sequencing was performed by Microsynth or in‐house.

### 2.5 Omni‐ATAC‐Seq

Omni‐ATAC‐Seq was performed according to [54] without DNAse treatment. After transposition, samples were further processed at the Cologne Center for Genomics (CCG). The purified DNA after the transposase reaction was amplified using the NEBNext High‐Fidelity 2× PCR MasterMix (New England Biolabs, Frankfurt am Main, Germany) and Nextera DNA 96 UDIs (Illumina, Berlin, Germany). The PCR program consisted of an initial incubation at 72 °C for 5 min followed by denaturation at 98 °C for 30 s, and subsequently 8 cycles of 10 s at 98 °C, 30 s at 68 °C and 72 °C at 1 min. This was followed by double‐sided bead purification to remove primer‐dimers and large fragments >1000 bp. After library validation and quantification (Agilent Tape Station), equimolar amounts of library were pooled. The pool was quantified by using the Peqlab KAPA Library Quantification Kit and the Applied Biosystems 7900HT Sequence Detection System. The pool was sequenced on an Illumina NovaSeq6000 sequencing instrument with a PE100 protocol aiming von 50 million clusters per sample.

Paired‐end reads were trimmed with trimmomatic (v0.38) [55] to remove adapter sequences and low‐quality bases. Reads were cropped to a maximum length of 90 bp to remove any remaining adapter sequence. Dovetailed alignment to the human reference genome (hg38) was done using bowtie2 (v2.4.1) [56], allowing a maximum insert size of 2000. Duplicated and mitochondrial reads were discarded. The 5′ ends of the reads were shifted with deepTools alignmentSieve (v3.4.3) [57] to match the center of the Tn5 binding site.

Libraries were downsampled to an equivalent size with Picard DownsampleSam (v2.10.6) [58]. Peak regions were then called for each sample using MACS2 (2.1.2) [59] with parameters ‐‐nomodel ‐‐shift −100 ‐‐extsize 200. Peaks within ENCODE blacklisted regions were excluded [60]. Downstream analyses were performed with R/Bioconductor packages. Differential accessibility analysis was performed for consensus peaks using edger (v3.42.4) [61], excluding low signal‐abundance regions (log_2_ CPM < −1.5) and regions detected only in one replicate. Regions <500 bp apart were merged with the csaw R package (v1.33.0) [62], restraining the maximal merged window to 5 kb. The most significant region was used as a statistical representation when merged. FDR‐adjusted *P*‐values were then computed, considering accessibility changes with an FDR below 0.01 as significant.

Peaks were annotated with ChIPseeker (v1.36.0) [63], using the TxDb.Hsapiens.UCSC.hg38.knownGene database [64]. Regions ± 3 kb around the transcriptional start site (TSS) were considered to be gene promoters. Distal and proximal enhancers were annotated according to ENCODE cell type‐agnostic cis‐regulatory elements (cCRE). The R package clusterprofiler (v4.8.2) [65] was used to perform over‐representation analysis of genes associated with differentially accessible regions.

### 2.6 RNA‐sequencing

Cells were seeded in 6‐well plates and grown for 48 h to reach 70–80% confluence. Cells were harvested, and the RNA isolated using NucleoSpin Mini kit (Macherey‐Nagel, Düren, Germany). Samples were processed at the Cologne Center for Genomics (CCG). Libraries were prepared using the Illumina^®^ Stranded TruSeq^®^ RNA sample preparation kit. ERCC RNA Spike‐In Mix 1 (Thermo Fisher Scientific) was added to the samples before library preparation. Library preparation started with 1 μg total RNA. After poly‐A selection (using poly‐T oligo‐attached magnetic beads), mRNA was purified and fragmented using divalent cations under elevated temperature. The RNA fragments underwent reverse transcription using random primers. This was followed by second strand cDNA synthesis with DNA Polymerase I and RNase H. After end repair and A‐tailing, indexing adapters were ligated. The products were then purified and amplified (15 PCR cycles) to create the final cDNA libraries. After library validation and quantification (Agilent Tape Station), equimolar amounts of library were pooled. The pool was quantified by using the Peqlab KAPA Library Quantification Kit and the Applied Biosystems 7900HT Sequence Detection System. The pool was sequenced on an Illumina NovaSeq6000 sequencing instrument with a PE100 protocol aiming at 60 million clusters per sample.

Paired‐end reads were trimmed with trimmomatic (v0.38) [55] to remove adapter content and bad quality bases. Reads were aligned to the human reference genome (hg38) and reads‐per‐gene were quantified using star (v2.5.2b) [66]. Downstream analysis was performed with R/Bioconductor packages. A linear model‐based approach, limma R package (v3.54.2) [67], was used to identify differentially regulated genes across ARID1A expression and deficiency. The gage R package (v2.48.0) [68] was used to perform gene set enrichment. Reference gene sets, such as Hallmark, KEGG, and Gene Ontology, were downloaded from MSigDB (v7.0) [69]. In both analyses, an adjusted *P*‐value below 0.05 was considered significant.

For alternative splicing analysis, reads per exon were quantified using the DEXSeq R package (v1.44.0) [70]. Exons that were quantified in more than 3 samples were selected for further analysis. Then exon‐level differential analysis was performed using the DiffSplice function from limma.

### 2.7 Co‐immunoprecipitation (Co‐IP)

HBLAK and UROtsa were gently lysed in IP‐1 (20 mm HEPES, pH 7.4; 150 mm NaCl; 0.5% NP‐40; 2 mm EDTA; 1 complete mini tablet/10 mL; 1× phosphatase inhibitor). After pre‐clearing, co‐immunoprecipitation was performed using 5 μL ARID1A (D2A8U) (12 354; Cell Signaling) antibody or 1 μg rabbit IgG isotype control (DA1E) (3900; Cell Signaling). Three biological replicates per condition (ARID1A specific antibody/IgG control in both cell lines) were separated by SDS/PAGE. Three gel pieces were excised per gel lane and digested with Trypsin as described previously [71]. After lyophilization, the peptides were resuspended in 3% formic acid (FA)/5% acetonitrile (ACN) and loaded onto a nanoLC system (RSLCnano; Thermo Scientific, Germany). First, the peptides were trapped on a precolumn (Acclaim PepMap100, C18, 5 μm, 100 Å, 300 μm i.d. ×5 mm; Thermo Scientific) for 10 min and then separated on an analytical column (Easyspray 50 cm column (ES803); 45 °C; Thermo Scientific) using a 90 min gradient (0–10 min: 5% buffer B (buffer A: 0.1% FA; buffer B: 80% ACN, 0.1% FA), 10–55 min: 10–35% buffer B, 55–65 min: 35–50% buffer B, 65–66 min: 50–99% buffer B, 66–70 min: 99% buffer B, 70–71 min: 99%–5% buffer B, 71–90 min: 5% buffer B). Spray voltage: 2 kV; capillary temperature: 250 °C.

The eluted peptides were then analyzed on a Q Exactive plus mass spectrometer (Thermo Scientific) in data‐dependent mode. Full MS settings: resolution: 70 k; AGC target: 3e6; maximum injection time: 100 ms; range: 300–1650 m/z. dd‐MS2settings: resolution: 17.5 k; AGC target: 2e5; maximum injection time: 110 ms; isolation window: 1.8 m/z; normalized collision energy: 27; precursor fragmentation: top 10. dd‐MS settings: minimum AGC target: 5e2; dynamic exclusion window: 10s; only 2+−5+ peptides.

Analysis of the raw data was performed in maxquant (v1.6.10.43) with the built‐in Andromeda search engine [72] and default settings. The spectra were searched against a human UniProt fasta file (01/2020; only reviewed and canonical sequences). Specific settings were: Specific protease: Trypsin (with two missed cleavages allowed); fixed modification: Carbamidomethylation (on Cysteine residues); variable modifications: Oxidation (on Methionine residues) and N‐terminal protein acetylation. Quantification was performed using the label‐free quantitation algorithm from MaxQuant.

The proteinGroups.txt result files (File S1) from MaxQuant search were then individually analyzed for both cell lines using perseus (v1.6.14.0; [73]). The LFQ intensities from all biological replicates were set as the main columns. The entries were filtered for reversed hits, contaminants, and “only identified by site” protein identifications, which were all removed. A protein had to be identified with a minimum of two unique peptides. The individual biological replicates were grouped into their corresponding experiments (ARID1A and IgG) and the LFQ intensities were then transformed by applying log_2_. The final dataset included only proteins that were identified in all replicates in at least one group (min 3 in one group). The data was then subjected to value imputation based on normal distribution (Perseus default settings; File S1) and a two‐sample test was applied. The resulting data was used for Fig. 3A.

### 2.8 Colony formation assay (CFA)

1000 T24, 1500 UROtsa, or 3000 JMSU‐1 were seeded in a 6‐well and grown for 10–14 days with appropriate medium exchanges. Grown cells and colonies were washed with PBS, stained with crystal violet, imaged, and analyzed with imagej v1.53m.

### 2.9 CellTiterGlo assay

Transfected cells were grown for 24 h, then seeded in appropriate 96‐Well plates with 2500 cells for UROtsa and 2000 cells for T24 per well. The CellTiter‐Glo assay (Promega) was performed according to the manufacturer's instructions 48 and 72 h after seeding.

### 2.10 Ionizing radiation (IR) source and setup

The setup was as described [74]. Briefly, cells in cell culture dishes with medium were irradiated using 6 MV X‐ray beams from a medical patient linear accelerator. The distance between the source and buildup was 100 cm, and 3 cm of water‐equivalent material was placed on the opposite surface of the cell culture plates to allow backscatter.

### 2.11 Radiation‐induced cell cycle arrest

Cells were seeded at 70% confluency. On the following day, cells were irradiated with 8 Gray as described above. At the indicated times, cells were harvested, washed with PBS containing 1% FCS, resuspended in 1 mL cold PBS, and fixed by adding 4 mL 100% ethanol while vortexing. Before staining, cells were washed three times with PBS containing 1% FCS and then stained using a propidium iodide staining solution (0.2% V/V RNAse A (50 mg·mL^−1^) and 5% V/V propidium iodide (1 mg·mL^−1^) in PBS with 1% FCS). Cells were measured using a BD Canto II (BD, Heidelberg, Germany) and results were analyzed with flowjo v10.8.1.

### 2.12 qPCR splicing

RNA was isolated using the NucleoSpin Mini kit (Macherey‐Nagel, Düren, Germany) and transcribed using a Reverse Transcription System (Promega, Walldorf, Germany). After transcription, RT‐PCR was performed at 60 °C with iTaq Universal SYBR Green Supermix (Bio‐Rad, Feldkirchen, Germany).

### 2.13 Statistics

All data was considered significant if *P* < 0.05. A *t*‐test was performed for CFA, cell cycle distribution after DNA damage, and splicing analysis.

## 3 Results

### 3.1 Truncations are the most frequent mutation type of *ARID1A* in bladder cancer patients

To understand the consequences of the high prevalence of *ARID1A* mutations in cancer, we based our analysis on MSK (Memorial Sloan Kettering) cancer panel data as of 2022, containing 1659 bladder cancer samples from 1244 patients [23]. We found that *ARID1A* mutations were distributed throughout the coding region regardless of domains or post‐translational modifications across patients (Fig. 1A). Utilizing methodology partially based on [75], two cancer hotspots (X911 and G2087) were marked as statistically significantly recurrent mutations across multiple cancers. However, the most frequent mutation in the MSK bladder cancer cohort was affecting the codon for Q515, which was observed in 7 patients. While only one patient harbored a missense mutation (Q515F), all others carried nonsense mutations at Q515, resulting in premature termination of translation. Indeed, the overall most frequent mutations were truncating (77%) with missense mutations (15%) being the second most frequent (Fig. 1B). Other genetic alterations of *ARID1A*, including in‐frame mutations and deletions, were rarely found in the cohort. Interestingly, missense and truncating mutations are frequent in the top 10 mutated genes in bladder cancer from the MSK panel (Fig. 1C). An exception to this is *TERT* mutations, which are almost exclusively located in the promoter region, facilitating re‐expression of the gene. *FGFR3* and *PIK3CA* predominantly display missense mutations, consistent with their oncogenic functions. Among these 10 genes, *ARID1A* shows the highest level of truncating mutations in this cohort.

As bladder cancer is a highly heterogeneous disease [76], we decided to use four different cell line models to mimic this heterogeneity. However, we acknowledge that models cannot entirely depict the spectrum of normal bladder cells to malignant and metastatic bladder cancer. Therefore, we generated *ARID1A* knockout (UROtsa, normal; and T24, malignant), knockdown (HBLAK, normal), and re‐expressing urothelial bladder cell lines (JMSU‐1, malignant, metastatic). These models were subsequently used for multidimensional OMIC approaches, including genomic, transcriptomic, and interactome analyses (Fig. 1D). The transcriptomes of several independent clones in all four cell models were measured using mRNA‐Seq. The interactome studies were carried out in the two normal‐like cell lines. Genomic studies were performed with the normal‐like UROtsa cells, and functional studies with up to three of the model cell lines. The focus was on UROtsa cells, as we hoped to understand more about cancer progression by studying this normal‐like model.

Knockout clones were generated using CRISPR/Cas9 with different guide RNAs binding to distinct exons of *ARID1A* (Table S1). Single‐cell clones of UROtsa and T24 were selected, and the mutation of *ARID1A* was verified by Sanger Sequencing (Fig. 1E; Fig. S1). Shown here is the insertion of a single nucleotide in the UROtsa clone #5 (generated with gRNA3), which is predicted to result in a truncation due to a frameshift. In addition to sequencing, ARID1A protein expression was analyzed by western blotting (Fig. 1F; Fig. S2). UROtsa clones generated from gRNA3 are shown, of which clones #29 and #31 did not display ARID1A protein expression. Together with clone #5 (gRNA3), clone #13 (gRNA3), and clone #14 (gRNA2), they were used for subsequent analysis after validation at both the genetic and protein levels (Figs [Link], [Link]). HBLAK cells, which express wildtype ARID1A, were treated with siRNA against ARID1A, mediating a knockdown of up to 73% on the transcript level and a decrease of protein in western blot analysis (Fig. S2). We re‐expressed wildtype ARID1A in the transformed JMSU‐1 cells (Fig. S2), which carry a homozygous frameshift *ARID1A* mutation that prevents expression [53].

### 3.2 Impaired cell proliferation and deregulation of DNA repair and stress‐related processes upon loss of ARID1A

**Fig. 2:**
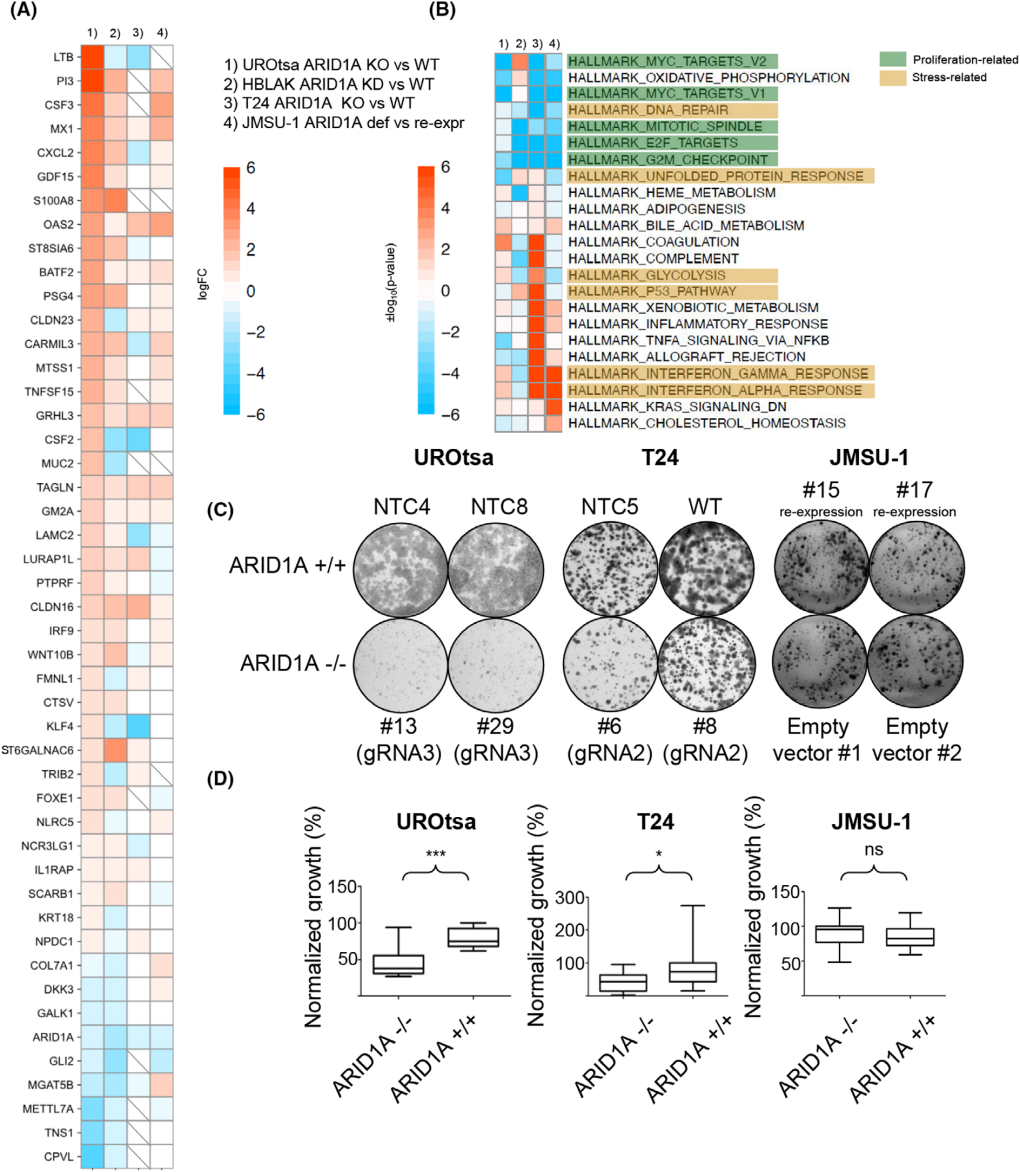
ARID1A‐deficient bladder (cancer) cells show impaired proliferation and deregulated stress‐related pathways. (A) Heatmap of 47 genes concurrently significantly deregulated in UROtsa and HBLAK cells upon ARID1A knockout and depletion, respectively. The data are extracted from our mRNA‐Seq data sets (UROtsa (*n* = 5 per group); other (*n* = 3 per group)) and compared across all four cell lines generated in this study (note that always ARID1A deficient vs. proficient data sets were compared as indicated in this panel, the same setting was used for panel B); (B) Hallmark gene set enrichment for benign and cancerous bladder cell lines. Color code represents the enrichment significance with positive values representing upregulation and negative values downregulation; (C) Colony Formation Assay (CFA) of UROtsa (*n* = 5 per group; ARID1A KO vs WT), T24 (*n* = 4 per group; ARID1A KO vs. WT), and JMSU‐1 (*n* = 5 per group; ARID1A re‐expression vs. mutated WT), representative images of three independent experiments are shown. (D) boxplots with whiskers min to max of the CFAs from C for UROtsa (*n* = 5), T24 (*n* = 4), and JMSU‐1 (*n* = 5), *t*‐test, ns, not significant, *P < 0.05, **P < 0.01, ***P < 0.001.

To understand the consequences of ARID1A deficiency, we evaluated the transcriptional landscape using mRNA‐Seq. Including all models generated in this study, we were able to examine the effect of ARID1A deficiency on normal‐like and malignant urothelial bladder cell lines. Of note is that in the UROtsa, T24, and JMSU‐1 cells, single clones were established from both the wildtype and the manipulated clones, while in the HBLAK cells, KD pools were used. Thus, in the former three cell lines, long‐term effects are likely to predominate, while in the latter, short‐term effects are analyzed. The mRNA‐Seq experiments were carried out in 5 clones per group (*ARID1A* KO and *ARID1A* WT, respectively) for UROtsa and three samples per group for the other three cell lines. UROtsa showed more than 2400 significantly deregulated transcripts with a fold change greater than 1.5 when wildtype clones were compared to *ARID1A* knockout clones (GEO dataset GSE271301, File S2). In HBLAK cells, more than 360 differentially expressed genes were observed upon *ARID1A* knockdown (GEO dataset GSE271301, File S3). In the malignant models, fewer significantly deregulated transcripts were measured, with 129 for JMSU‐1 and only one for T24 with a fold change greater than 1.5 and an adjusted *P*‐value below 0.05 (GEO dataset GSE271301, [Link], [Link]). In the two normal‐like models UROtsa and HBLAK, the overlap of concurrently deregulated transcripts was 47 (Fig. 2A). Analyzing the fold change of these 47 transcripts in all four cell line models regardless of their *P*‐values, they presented several genes that were regulated in the same direction in the absence of ARID1A. A small group of genes, including *MX1*, *OAS2*, *BATF2*, *GRHL3*, *TAGLN*, *GM2A*, *CLDN16*, *IRF9*, and *ST6GALNAC6*, appeared to be activated in all four cell lines in ARID1A‐deficient cells. *MX1*, *OAS2*, *BATF2*, and *TAGLN* also displayed positive regulation in the TCGA 2017 dataset in ARID1A negative expression value patients, while there were no measurements for GM2A, CLDN16, IRF9, and ST6GALNAC6 (Fig. S3). This comparability of our models to the TCGA datasets argues for realistic models, which mimic cancers closely. *TAGLN* and *GRHL3* displayed similar fold change increases across all four cell lines, hinting at a possible direct regulation by ARID1A. *PI3*, *CSF3*, and *TNFSF15* transcripts were strongly upregulated in UROtsa, HBLAK, and JMSU‐1 ARID1A‐deficient cells but could not be detected in T24 cells. Expression of *S100A8* was strongly induced in both normal‐like cell lines but could not be measured in the tumor cell lines. If present, *ARID1A*, *GLI2*, *METTL7A*, and *TNS1* showed negative regulation on the transcriptome level in ARID1A‐deficient cells. Thus, a set of genes was deregulated comparably across different ARID1A‐expressing or ‐deficient cell lines.

A gene set enrichment analysis was performed to identify processes and functions that were affected by manipulating ARID1A expression in the different cell lines. ARID1A‐deficient cells revealed significantly fewer transcripts of genes annotated to cell proliferation, which include terms such as MYC targets, E2F targets, mitotic spindle, and G2M checkpoint. For JMSU‐1, the data from the wildtype cells, in which *ARID1A* is mutated, were compared to the data from the cells that re‐express ARID1A. Thus, in all models, the same directionality is analyzed, i.e., the changes from no or low levels of ARID1A to high ARID1A levels (Fig. 2B). Moreover, different stress‐associated pathways were deregulated, such as DNA repair, interferon response, glycolysis, p53 pathway, and unfolded protein response. While proliferative pathways were typically downregulated, stress‐associated processes did not show uniform regulation across the models. For example, the loss of ARID1A in T24 tumor cells led to strong activation of most of these pathways, while they were only weakly affected in the normal‐like cell lines. We noted that the absence of ARID1A in both T24 and JMSU‐1 tumor cells strongly induced interferon (IFN) type I and II pathways, but less prominently in the two normal‐like lines (Fig. 2B). Genes associated with allograft rejection were upregulated in T24 and JMSU‐1 tumor cells but downregulated in both normal‐like models in the absence of ARID1A. Interestingly, there was strong downregulation of the oxidative phosphorylation in all models but HBLAK.

To assess the functional consequences of the deregulation of proliferation‐associated pathways observed in the mRNA‐Seq data sets, we conducted colony formation assays. The KO of *ARID1A* in UROtsa and T24 significantly impaired cell proliferation (Fig. 2C,D). We noticed a reduction in both colony size and number in the two cell lines. To test whether reduction versus loss of ARID1A impacts cells differently, we performed *ARID1A* KD studies in T24 and UROtsa cells and measured cell proliferation using CellTiterGlo assays to determine cellular ATP levels. Our knockdown was efficient (Fig. S4A), yet we could not observe a major change in proliferation (Fig. S4B). Both cell lines showed only little change compared to a control siRNA.

In contrast, cell proliferation was not affected in JMSU‐1 upon ARID1A re‐expression (Fig. 2C,D). We noticed the strongest effect in the normal‐like UROtsa cells, an effect in the tumorigenic T24 cells, while the ARID1A‐deficient tumorigenic JMSU‐1 cells seem to have fully adapted to the lack of ARID1A.

### 3.3 ARID1A contributes to alternative splicing and translational regulation

**Fig. 3:**
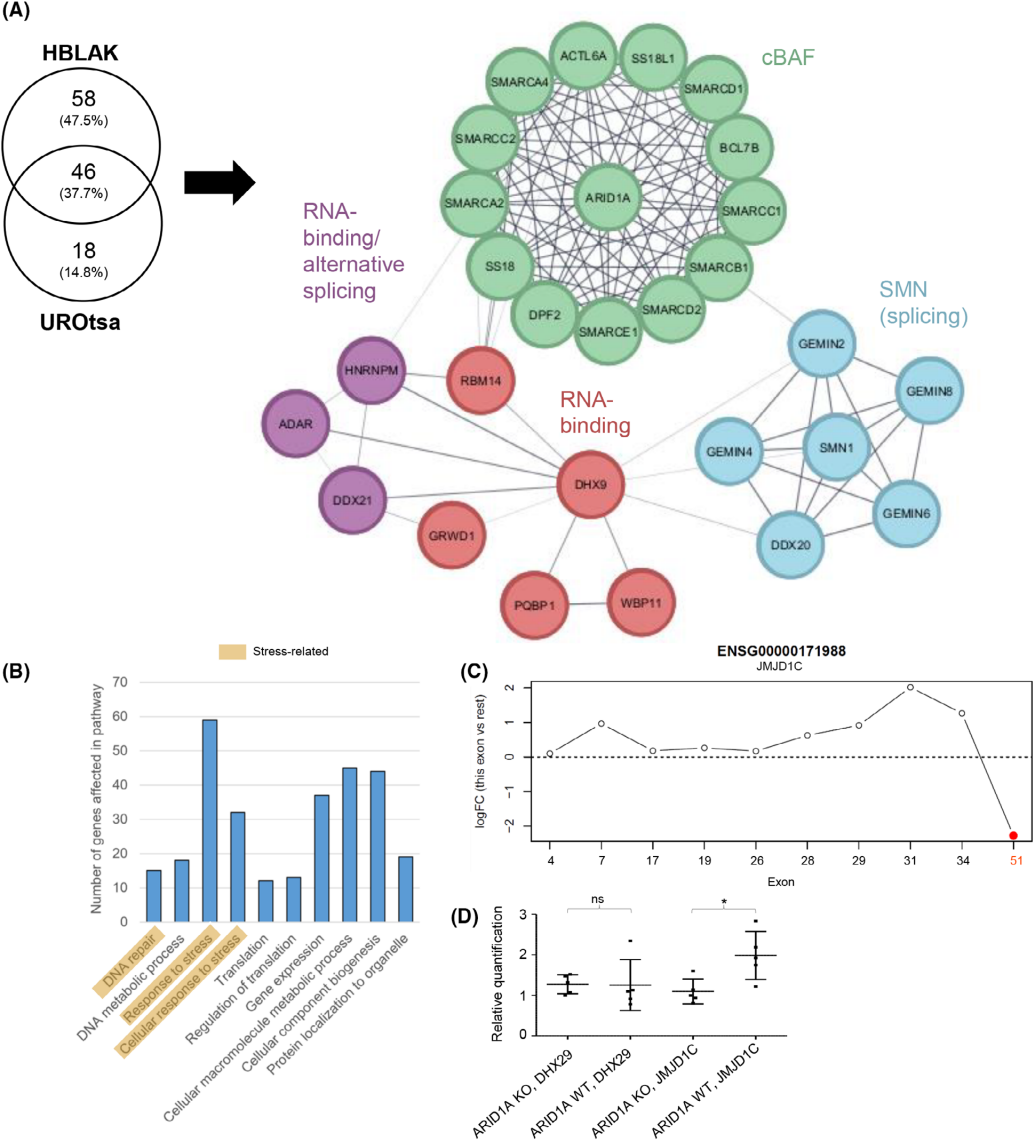
ARID1A interactome and splicing effects. (A) Consensus interactome of 10× enriched interaction partners of ARID1A co‐immunoprecipitation (Co‐IP) using the benign HBLAK and UROtsa cells (*P* < 0.05), single nodes were deleted for better visuality; all enriched proteins can be found in File S1. (B) Differential splicing analysis based on our mRNA‐Seq data, annotated to gene set enrichment pathways. Single nodes were deleted for clarity. (C) Alternative splicing of JMJD1C in UROtsa, the red dot marks significant alteration in ARID1A KO vs. WT (*n* = 5); (D) *t*‐test of RT‐qPCR measurement of possible alternatively spliced factors in ARID1A KO UROtsa (mean with SD of triplicates of *n* = 5 per group). ns, not significant, *P < 0.05. Figure was designed using cytoscape (v3.10.2).

To understand more about the molecular functions of ARID1A in the normal urothelium, we performed a co‐immunoprecipitation (Co‐IP) experiment followed by mass spectrometry in both HBLAK and UROtsa cells (Fig. 3A). The proteins enriched more than 10‐fold in the two cell lines were compared. We observed the expected interaction with cBAF complex members such as SMARCB1 and SMARCA4 (Fig. 3A). In addition, interactions with ribosomal and SMN complex proteins were measured, suggesting a potential involvement of ARID1A/cBAF complexes in the regulation of translation and splicing. Indeed, analyzing our mRNA‐Seq data for alternative splicing in our UROtsa model revealed more than 8000 alternatively spliced transcripts with a *P*‐value below 0.05 (File S6). After removing ambiguous transcripts and multiple entries for the same loci and genes, 2868 unique loci were significantly altered (File S6). Utilizing a GSEA analysis, we found genes associated with translation and gene expression pathways to be impacted by alternative splicing in ARID1A‐deficient cells (Fig. 3B). Also affected were stress‐related pathways, including DNA repair and cellular response to stress, as well as metabolic processes involving macromolecules or DNA. Together, we were able to reveal a possible direct interaction of ARID1A/cBAF with splicing‐associated factors and a potential involvement in translation seen both directly and via alternative splicing.

To validate this finding, we tested two candidates, *DHX29* and *JMJD1C*, for alternative splicing by RT‐qPCR. *JMJD1C* was chosen because exon 51 displayed significant alternative splicing in our mRNA‐Seq experiments with an underrepresentation upon ARID1A loss (Fig. 3C). RT‐qPCR confirmed the reduced expression of exon 51 in the *ARID1A* KO cells, while no significant regulation for *DHX29* was observed (Fig. 3D).

### 3.4 Reduced chromatin accessibility in the absence of ARID1A

**Fig. 4:**
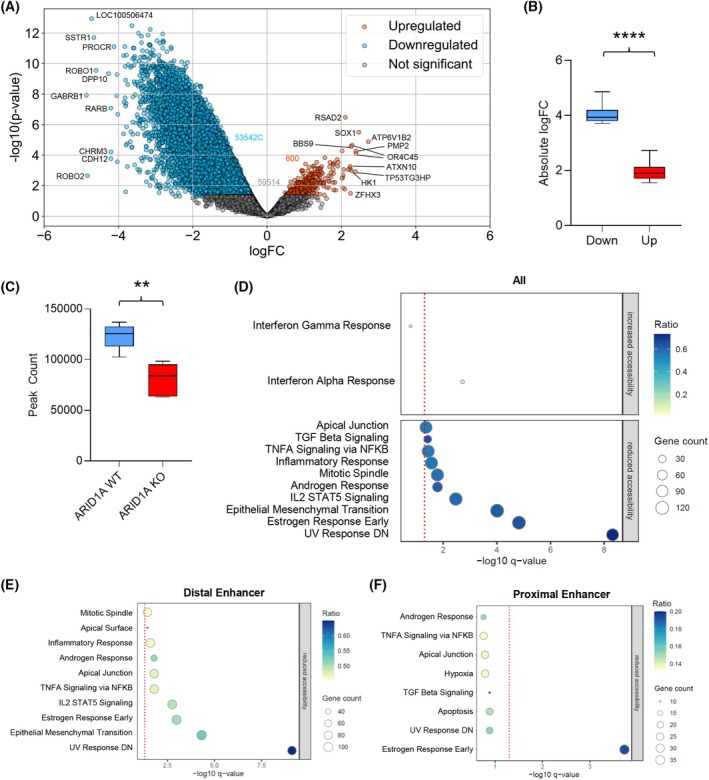
Absence of ARID1A leads to less accessible chromatin. (A) Differentially accessible chromatin in UROtsa ARID1A KO vs. WT cells (*n* = 5 per group) with top regulated regions indicated. Colored dots represent significantly altered chromatin sites (*P* < 0.05); (B) boxplot with whiskers min to max and *t*‐test of absolute logFC comparison of the top 50 regulated loci ****P < 0.0001; (C) boxplot with whiskers min to max and *t*‐test of the peak count from the Omni‐ATAC‐Seq, *n* = 5 per group, **P < 0.01; (D) All differentially accessible chromatin regions of UROtsa ARID1A KO vs. WT annotated to the Hallmarks gene sets, the dotted red line represents *q* = 0.05; (E) Top differentially accessible distal enhancers of UROtsa ARID1A KO vs. WT annotated to the Hallmarks gene sets, the dotted red line represents *q* = 0.05; (F) Top differentially accessible proximal enhancers of UROtsa ARID1A KO vs. WT annotated to the Hallmarks gene sets, the dotted red line represents *q* = 0.05.

As part of a chromatin remodeler complex, we aimed to determine the effects on chromatin accessibility upon loss of ARID1A. Therefore, we performed Omni‐ATAC‐Seq using the UROtsa model. As expected, chromatin was less accessible in ARID1A‐deficient UROtsa cells, suggesting an overall denser chromatin structure. Comparing 113,656 sites between ARID1A‐deficient and expressing cells, almost half (53542) became less accessible upon ARID1A depletion, while only 600 sites appeared more accessible (Fig. 4A, GEO dataset GSE271587, File S7).

Focusing on the top 50 regulated regions, a significant difference in fold change was apparent, showing greater fold changes for less accessible sites (Fig. 4B). The weakest fold change of the top 20 less accessible regions (*SHISA6*, logFC = −3.6) was still greater than the strongest fold change of the more accessible regions (*ATP6V1B2*, logFC = 2.7). The number of accessibility peak regions per clone was significantly lower in *ARID1A* KO clones compared to WT cells, further signifying the impact of ARID1A on chromatin architecture (Fig. 4C).

Examining the top 10 strongest chromatin accessibility changes, two genes known to impact the transcriptional landscape of cells were found: *CDYL* and *SOX1*. While *CDYL* showed less accessible chromatin mostly on the intron level, the distal intergenic regions of *SOX1* were more accessible. Showing less accessible chromatin on the intron level, *CDYL* transcripts were slightly increased in UROtsa ARID1A KO vs. WT cells (FC = 1.119; adjusted *P*‐value = 0.15, GEO dataset GSE271587). While we found more accessible *SOX1* intergenic regions, the transcripts were not measured in RNA‐Seq [77, 78]. Moreover, *ROBO1* (at intron and distal intergenic regions) and *ROBO2* (at introns) were less accessible, the encoded proteins affecting the Notch signaling pathway and chemotaxis. Indeed, *ROBO1* transcripts were significantly downregulated (FC = 0.711; adjusted *P*‐value 0.01; GEO dataset GSE271587). Neither mentioned gene above showed alternative splicing (File S6).

A gene set enrichment analysis was performed for all genomic regions with altered accessibility based on the Hallmark gene set [69] (Fig. 4D). Genes linked to the IFN alpha (*CD47*, *ISG20*) and gamma (*JAK2* and *VCAM1*) responses were associated with less dense chromatin, consistent with increased expression (Fig. 2B).

Lesser accessible regions were annotated to genes linked to cell cycle progression (for example CDK1 and CDC42), DNA damage response (MGMT, ATXN1), and epithelial‐mesenchymal transition (like ADAM12, COL16A1). Strikingly, functionally enriched differential accessible regions, but not differentially accessible regions in general, were frequently observed at sites in introns and distal enhancers, while promoters or exons were affected less (Fig. 4F; Fig. S5). Among functionally enriched enhancers, distal enhancers were more affected than proximal enhancers (Fig. 4E,F). Distal enhancers exhibited less accessible chromatin in regions annotated to processes associated with immune signaling, such as the IL2– STAT5 pathway, cell cycle processes like the mitotic spindle, epithelial‐mesenchymal transition (EMT), and DNA damage response.

### 3.5 ARID1A‐deficient cells show an impaired G2/M checkpoint after DNA damage

**Fig. 5:**
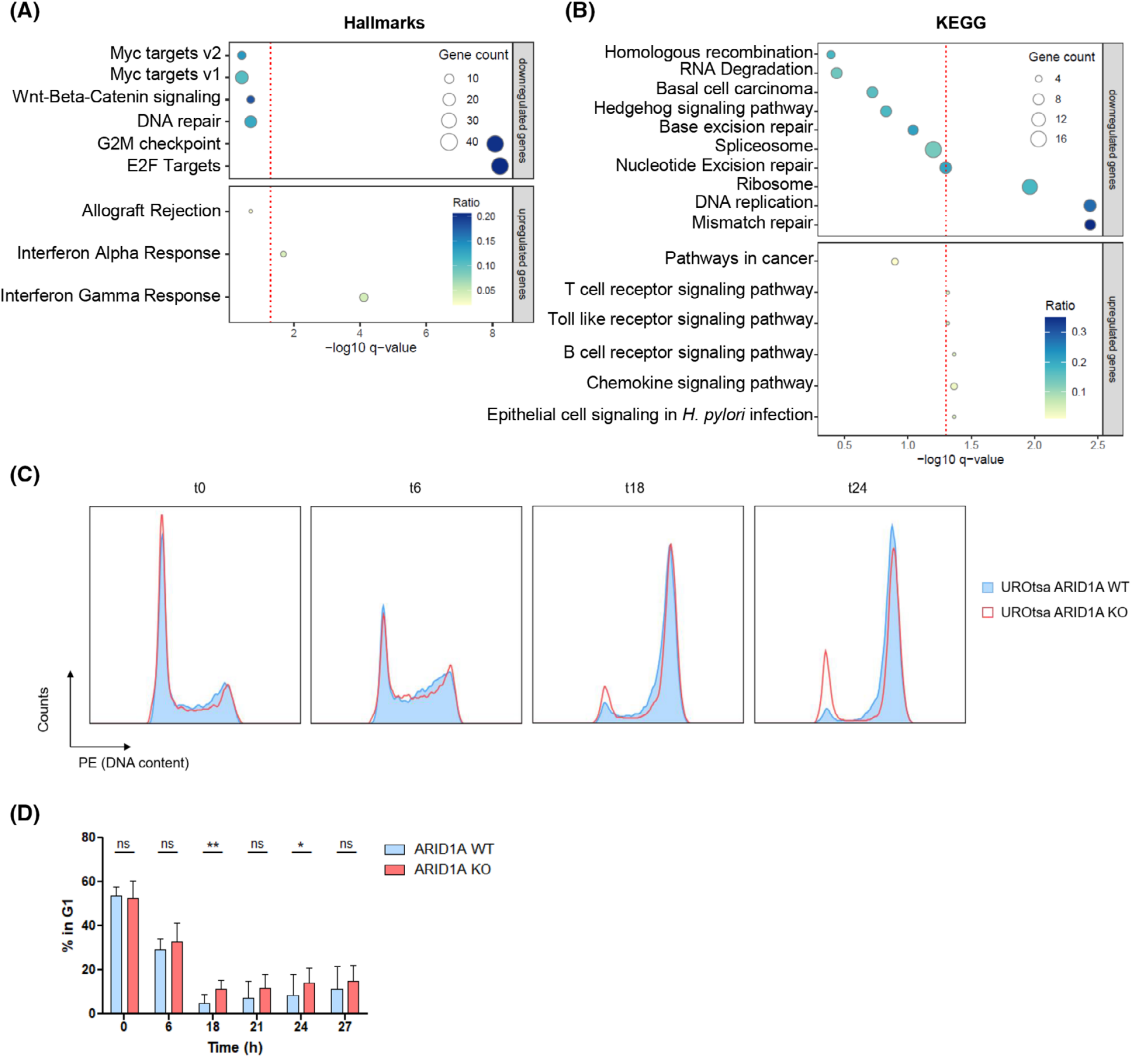
ARID1A‐deficient UROtsa display an impaired G2/M checkpoint. (A) Consensus of Omni‐ATAC‐ and RNA‐Seq annotated to the Hallmark gene set; the dotted red line represents *q* = 0.05. (B) Consensus of Omni‐ATAC‐ and RNA‐Seq annotated to the KEGG gene set; the dotted red line represents *q* = 0.05. (C) UROtsa cells were irradiated with 8 Gray and cultivated further for the indicated times. DNA content was analyzed using flow cytometry. Count normalized cell cycle histograms are shown. (D) Relative portion of cells in the G1 phase at the indicated time points after irradiation (*n* = 4 per group, three independent experiments, mean with SD), ns, not significant, *P < 0.05, **P < 0.01.

Intending to describe the impact of ARID1A on bladder cancer development, we correlated our datasets from the Omni‐ATAC‐Seq and the RNA‐Seq experiments in the UROtsa model (File S8). Effects were annotated to the Hallmark [69] and KEGG [79] gene sets (Fig. 5A,B). We observed a highly significant downregulation of genes associated with the terms G2/M checkpoint and E2F targets in the Hallmark gene set and an upregulation of the interferon pathway genes, consistent with the inhibition of proliferation as discussed before (Fig. 5B). This is further supported by the downregulation of the DNA replication gene set in the KEGG analysis (Fig. 5B). Moreover, the mismatch repair pathway was the most significantly downregulated KEGG gene set, a repair pathway that is particularly important in S and G2 cells and thus associated with proliferation. Despite being strongly regulated in the RNA‐Seq dataset, MYC targets no longer showed statistically significant deregulation when combining both Omni‐ATAC‐Seq and RNA‐Seq data.

As our genomic and transcriptomic analyses hinted towards a dysregulated cell cycle (control) and DNA‐damage pathways, we evaluated alterations in the cell cycle after DNA‐damage induction. UROtsa ARID1A WT (*n* = 5) and KO cells (*n* = 5) were irradiated with a high dose (8 Gray) of ionizing radiation, and the cells were harvested at different times. Non‐irradiated WT and KO cells displayed normal cell cycle distribution with about 50% of cells in G0/G1 (Fig. 5C). After irradiation, a steady buildup of G2/M cells could be observed over 24 h, indicating that these cells were proceeding less efficiently through mitosis. While KO cells began to re‐enter G1 by 18 and 24 h, WT cells stayed in G2/M. Minor differences were observed between the single clones; yet, the overall trend was comparable, showing significant differences in their behavior after DNA damage (Fig. 5D).

## 4 Discussion

Analysis of the MSK cancer panel data of 2022 [23] revealed *ARID1A* mutations to be foremost truncating, which leads to inactivation and protein loss through rapid degradation [15, 24, 80, 81, 82, 83]. Rapid degradation of truncated fragments is thought to be relevant as the C‐terminal BAF250_C domain of ARID1A is lost, which is hypothesized to be an important binding domain for other SWI/SNF subunits [20]. Additionally, the BAF250_C domain has been found to bind EZH2, a subunit of the PRC2 complex, an antagonist of SWI/SNF complexes [21]. Due to the significance of the C‐terminal region, we argue that the generated knockout clones represent truncating mutations the best.

Our mRNA‐Seq showed deregulation of interferon signaling with strongly induced pathways in both T24 and JMSU‐1 and less prominent deregulation in the two normal‐like cell lines UROtsa and HBLAK. Deregulated interferon signaling is frequent in cancers [84, 85]; an upregulation in ARID1A‐deficient cells may therefore either enhance or decrease these effects depending on the specific regulation on a cellular level. Additionally, we found allograft rejection to be deregulated on a transcript level, which may be tumorigenic, as increased allograft rejection is associated with high inflammatory processes, a common event in bladder cancer. This is consistent with other terms that are differentially regulated between normal‐like/benign and tumorigenic cells, including TNFα signaling and inflammatory response. All in all, broad deregulation of gene expression could be observed in response to altering ARID1A. We note that several are associated with tumorigenesis, including in particular proliferative, but also stress‐related, inflammatory, and DNA repair pathways.

In a colony formation assay, we tested whether the transcriptional deregulation of proliferation had an impact on the cells and found significant growth reduction in our *ARID1A* KO models T24 and UROtsa. We argue that the IFN type I response induction might also contribute to the reduced proliferation. Thus, in UROtsa and T24 cells, ARID1A is necessary for efficient proliferation.

A previous study reported that the knockdown of *ARID1A* in T24 cells resulted in increased proliferation [86]. To test whether reduction vs. loss of ARID1A impairs proliferation differently, we performed a knockdown experiment of our own. Unlike Cao *et al*. [86], our knockdown was efficient, and we could not observe major changes in proliferation. These findings indicate that the cell lines proliferate relatively normally with low levels of ARID1A, while complete loss is inhibitory. Nevertheless, a possible explanation is that the reduced versus lost ARID1A expression results in distinct phenotypes in these cells. Overall, the kind of alteration in ARID1A protein level, i.e., increased, decreased, or loss, but possibly also the distinct mutation as well as the combinations with mutations in other genes, may have specific consequences on cell proliferation. Reduced ARID1A expression may therefore be beneficial for the tumor while its loss has an inhibitory effect.

JMSU‐1 cells showed a strong IFN response and repression of several proliferation‐associated GO terms in the absence of ARID1A, which suggests that these tumor cells have adapted to cope with these potentially repressive effects. Upon re‐expression of ARID1A, no increase in proliferation was observed, despite the repression and activation of the IFN and the proliferative response, respectively. However, JMSU‐1 carries a *TP53* mutation (R280T) [87], which may be the driving force in this case, which cannot be restored by ARID1A reintroduction. Thus, we argue that the time during cancer development or progression may be crucial for the effects of the depletion of ARID1A and that early mutation, as is observed frequently [15], has the greatest impact on the cell. In conclusion, our findings suggest that ARID1A is important for cell proliferation by controlling many relevant genes.

We observed ARID1A deficiency impacting splicing and DNA repair on a transcriptomic level. By testing two candidates of alternative splicing, we found that whereas DHX29 is vital for the efficient translational initiation of mRNAs with structured 5’‐UTRs, the histone demethylase JMJD1C regulates various processes, including DNA double‐strand break repair and gene transcription and is deregulated in tumors [88, 89, 90]. We found *JMJD1C* to be significantly alternatively spliced, providing a further link between ARID1A and DNA repair processes.

As chromatin remodeler, we tested the accessibility of chromatin after *ARID1A* KO and found significantly less accessible chromatin, highlighting ARID1A's importance in decondensing chromatin. Decreased chromatin accessibility has also been observed for other cancer types such as colon and cervical cancer, arguing for a context‐independent function of ARID1A [91, 92]. Differentially accessible introns or distal intergenic regions may promote or inhibit translation, depending on whether they contain enhancers or negative regulatory elements. Additionally, the deregulation of introns can lead to alternative splicing [93]. Less accessible chromatin was measured for the transcriptional corepressor CDYL, which has been proposed as a positive regulator for the Polycomb repressor complex 2 (PRC2), an antagonist of SWI/SNF complexes [77, 78, 94, 95], showing regulation between these two antagonists. For *SOX1*, a more accessible region was found in the intergenic region. Commonly, the transcription factor SOX1 is downregulated in cancer due to promoter hypermethylation [96, 97, 98], however, the inhibition of SOX1 in glioma stem cells has been found to decrease proliferation *in vitro* [96], while overexpression of *SOX1* suppresses invasion and growth in lung cancer [98]. Therefore, this possibly links ARID1A deficiency to reduced invasiveness. Affecting the Notch signaling pathway and chemotaxis [99], ROBO1 and ROBO2 have been found less accessible post ARID1A loss, thus ARID1A‐deficient cells might be more motile and may evade chemical treatment better.

As our data indicated that the loss of ARID1A promotes the expression of interferon‐stimulated genes (ISGs), and limited accessibility and impaired expression of ISGs was found to correlate with poor immunotherapy response in ovarian clear cell carcinoma [21], we speculate that ARID1A‐negative tumors may be particularly sensitive to immunotherapy. Supporting this hypothesis, Shen *et al*. found reduced tumor burden and prolonged survival in mice after anti‐PD‐L1 antibody treatment of ARID1A‐deficient ovarian tumors [41].

Previous studies have found preferential positioning of cBAF complexes at enhancers [100]; however, while observing functionally enriched terms at enhancers, we did not observe a general co‐occurrence of cBAF complexes with enhancers. Generally, mutations in regulatory elements are frequent across all cancers, but bladder tumors show the highest percentage of mutated regulatory elements [101]. Changes in the accessibility of introns may lead to alternative splicing or intron retention [102], further amplifying the effects of ARID1A deficiency on the cells, and thus potentially contributing to tumorigenesis. After ionizing radiation, we found ARID1A‐deficient UROtsa cells to progress throughout the cell cycle normally, while ARID1A‐proficient cells arrested in G2/M. Therefore, we argue that ARID1A‐deficient UROtsa cells may have a less stable G2/M checkpoint, which results in at least partial arrest failure and premature re‐entry into G1 after irradiation damage. This deficit may drive cancer progression as DNA‐damaged cells continue proliferation. Li *et al*. also showed an impaired G2/M checkpoint in ARID1A‐deficient ES2 and HCT116 cells [103]. As a result, genetic instability is likely to increase because mutations may be inefficiently repaired due to the impaired G2/M checkpoint, possibly further facilitating tumorigenesis.

## 5 Conclusion

The main intent of our study was to better define the role of ARID1A in human bladder cancer. Therefore, we generated four cell line models as our basic tool and evaluated the effects of ARID1A manipulation on transcriptomic, interactomic, and genomic levels, as well as by functional analyses. We found *ARID1A* mutations to be mainly truncating mutations (77%) in patients with bladder cancer. To further advance our understanding of why ARID1A mutations arise in both early and late tumor development, we generated and evaluated three stable bladder (cancer) cell line models and additionally one transient model, i.e., two normal‐like (UROtsa, HBLAK) and two malignant (T24, JMSU‐1) models. Aiming to replicate truncations and the distribution of *ARID1A* mutations throughout the whole coding region, different guide RNAs were used to generate *ARID1A* knockouts in UROtsa and T24 cells.

*ARID1A* deficiency had severe impacts on several cellular pathways related to stress response and proliferation. We found strong upregulation of IFN pathways in the ARID1A‐deficient cells, especially in the malignant cell lines T24 and JMSU‐1. All cell lines showed decreased expression of DNA repair, mitotic spindle, MYC target (although upregulation in HBLAK cells), E2F target, and G2M checkpoint pathways. Testing the impact of the downregulated proliferation‐related pathways revealed impaired cell proliferation in our *ARID1A* knockout models (UROtsa and T24), but not in the *TP53‐mutated* JMSU‐1, whereas knockdown led only to minor proliferation alterations in UROtsa and T24 cells. This indicates that in early stages it may be beneficial for the tumor to mutate *ARID1A* for increased proliferation, whereas later other effects may be tumor supportive. We propose that testing for synthetic lethalities (*ARID1B* knockdown or BRG1/BRM degraders for example) may hold therapeutic promise.

A direct interaction with members of the SMN complex as well as a differential splicing analysis of our RNA‐Seq data revealed changes in the alternative splicing pattern of many genes upon ARID1A deficiency. The alternatively spliced genes belonged to stress‐related pathways including DNA repair, but also translational regulation. One of the tested and verified alternatively spliced candidates, JMJD1C, is a histone demethylase that regulates DNA double‐strand break repair and is involved in tumorigenesis [89], linking ARID1A once more to cancer.

We found that the deficiency of ARID1A had a strong effect on chromatin, leading to significantly less accessible chromatin in UROtsa cells. This was functionally especially apparent at distal enhancers and introns, but not promoters, where only the estrogen response was significantly regulated. The GSEA significantly altered chromatin regions at distal enhancers and introns were annotated to processes such as mitotic spindle, IL2 STAT5 pathway, EMT, or UV response, revealing once more the broad impact of a dysregulated chromatin remodeler.

Lastly, we proposed an impaired G2/M checkpoint for UROtsa cells with ARID1A KO, as these cells re‐entered G1 faster after DNA damage in comparison to ARID1A WT UROtsa cells, which accumulated in G2/M.

All in all, we found that ARID1A deficiency impacts several cellular processes that are relevant for tumor formation, including cell proliferation, EMT, cell cycle checkpoints, splicing, and immune response. While some processes are oncogenic, others show tumor‐suppressive character. The decreased proliferation upon ARID1A depletion is tumor‐suppressive, yet *ARID1A* is frequently mutated in early lesions already. We therefore hypothesize that mutating one allele of *ARID1A* may benefit the tumor, while a second hit would indeed decrease proliferation, harming tumor development. Losing the second allele might be tolerated or even beneficial at later stages of cancer when other key drivers such as *TP53* or *PTEN* are mutated. The second hit on *ARID1A* might support tumor growth and progression in other ways, for example, by increasing the genetic instability by interfering with efficient DNA damage repair. This is surmised from our cell cycle experiments combined with radiation‐induced damage, where ARID1A‐deficient cells did not arrest at the G2/M checkpoint but leaked into G1. Here, intervening in DNA‐damage pathways might be the key strategy for treatment.

## Conflict of interest

The authors declare no conflicts of interest.

## Author contributions

RK, SG: conceptualization. RMS, FK, SG, KB, FL, CP: methodology. GA, EC, MB: software and statistics OMICS. RMS, BL: original draft. RK, SG, BL: review and editing. SG, RK, BL: supervision. RMS: visualization. AG: additional experiments for revision. All authors have read and agreed to the published version of the manuscript.

## Supporting information


**Fig. S1.** Western blots of all used models in this study showing ARID1A deficiency or proficiency, respectively. (A) HBLAK treated with or without siRNA against ARID1A. (B) CRISPR/Cas9‐mediated KO of ARID1A in UROtsa, KO clones are marked in red. (C) ARID1A re‐expression in JMSU‐1. (D) CRISPR/Cas9‐mediated KO of ARID1A in T24.

**Fig. S2.** Sanger Sequencing results of generated T24 and UROtsa ARID1A KO. Each panel shows the respective clone, wildtype sequence and sequence of the KO. (A) T24 ARID1A KO gRNA1 KO#3. (B) T24 ARID1A KO gRNA2 KO#6. (C) T24 ARID1A KO gRNA2 KO#7. (D) T24 ARID1A KO gRNA2 KO#8. (E) UROtsa ARID1A KO gRNA2 KO#14; (F) UROtsa ARID1A KO gRNA3 KO#13; (G) UROtsa ARID1A KO gRNA3 KO#29; (H) UROtsa ARID1A KO gRNA3 KO#31.

**Fig. S3.** Translational regulation of genes found commonly regulated in UROtsa (*ARID1A* KO vs WT) and HBLAK (*ARID1A* KD vs WT) in TCGA 2017 cohort. Not all 47 significantly impacted genes were measured in the TCGA cohort. The median expression is shown for the median of negatively regulated ARID1A, positively regulated ARID1A, and overall regulation.

**Fig. S4.** The effect of siARID1A KD on UROtsa and T24 cells. (A) Western blot and Ponceau staining of an siRNA KD in UROtsa and T24 cells. B: Bar chart results of a CellTiterGlo assay of UROtsa and T24 treated with siRNA against ARID1A. Each clone was transfected twice and seeded in triplicates for the CellTiterGlo measurements. Measurements for 48–72 h were averaged and shown.

**Fig. S5.** Hallmarks GSEA of ARID1A KO vs WT UROtsa from an Omni‐ATAC‐Seq of (A) promoters, (B) exons, (C) exons and introns, (D) exons, introns, and 3′UTR.

**File S1.** ARID1A_interactome_HBLAK_UROtsa.xlsx.

**File S2.** DEG_UROtsa_ARID1A_KO_vs_NTC.xlsx.

**File S3.** DEG_HBLAK_ARID1A_KD_vs_WT.xlsx.

**File S4.** DEG_JMSU‐1_ARID1A_re‐expression_vs_defective_WT.xlsx.

**File S5.** DEG_T24_ARID1A_KO_vs_NTC.xlsx.

**File S6.** diffSplice_UROtsa_ARID1A_KO_vs_NTC.xlsx.

**File S7.** all_differentially_accessible_chromatin_ATAC‐Seq.xlsx.

**File S8.** ATAC‐Seq‐RNA‐Seq_integration_upSet_FDR0.05.xlsx.

**Table S1.** Oligos used in this study. Guide RNA (gRNA) sequences are underlined.

## Data Availability

Memorial Sloan Kettering (MSK) bladder cancer panel data 2022 [23] were downloaded from cbioportal [24, 25, 26]. The ATAC‐Seq datasets supporting the conclusions of this article are available in the Gene Expression Omnibus (GEO) repository, GSE271587, GEO Accession viewer (nih.gov). The RNA‐Seq datasets supporting the conclusions of this article are available in the Gene Expression Omnibus (GEO) repository, GSE271301, GEO Accession viewer (nih.gov). The mass spectrometry proteomics data have been deposited in the ProteomeXchange Consortium via the PRIDE [104] partner repository with the dataset identifier PXD054817. The results of Fig. S3 are based upon data generated by the TCGA Research Network: https://www.cancer.gov/tcga.
