# Supplementary figures and images for: Multidimensional OMICs reveal ARID1A orchestrated control of DNA damage, splicing, and cell cycle in normal‐like and malignant urothelial cells
Source: Mol Oncol. 2025 Apr 1;19(12):3784–805. doi: 10.1002/1878-0261.70019 (PMC12688176; doi:10.1002/1878-0261.70019)

**A****HBLAK**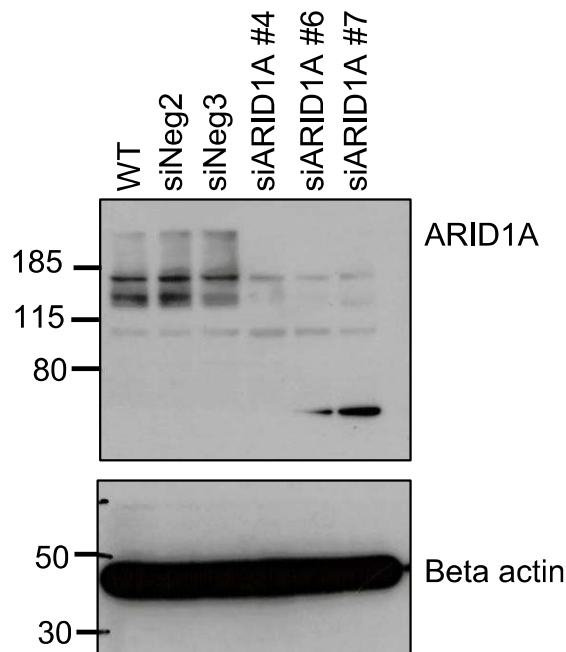**B****UROtsa**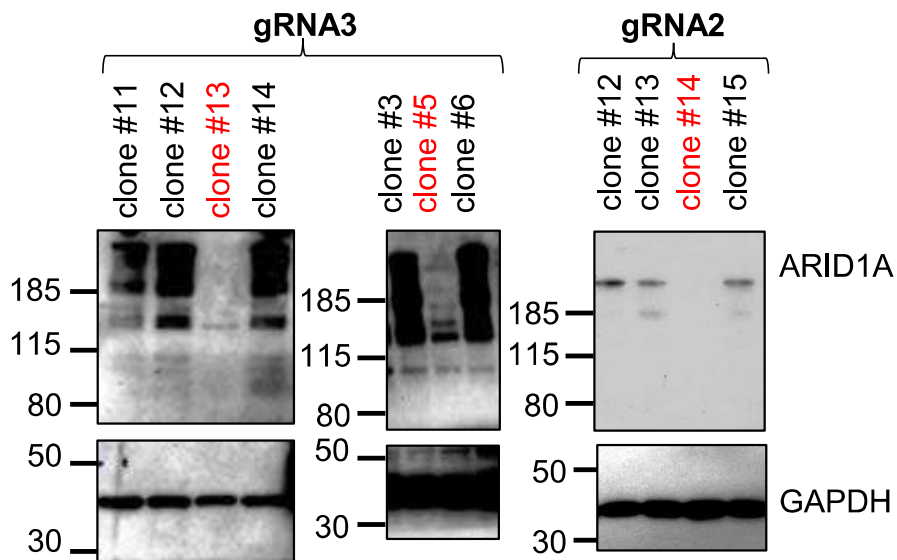**C****JMSU-1**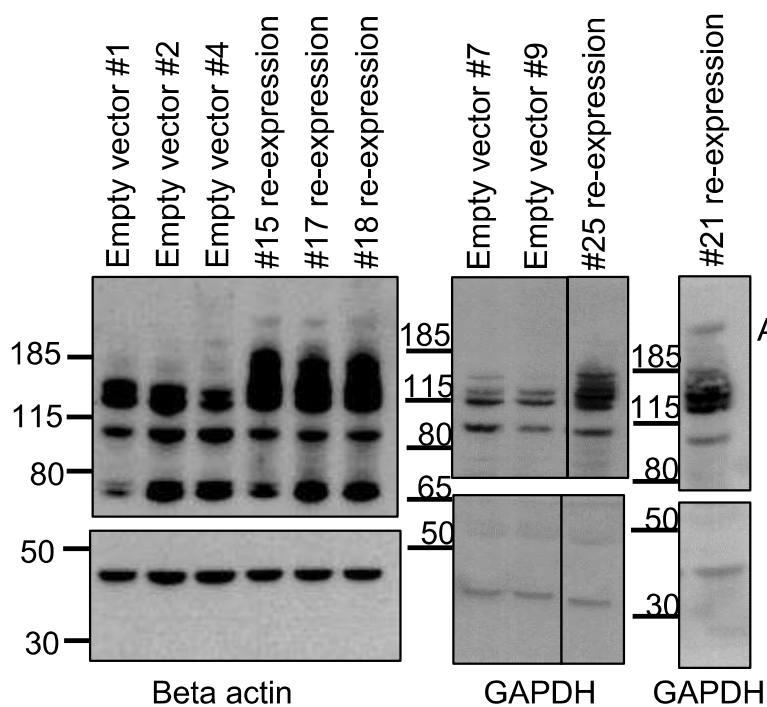**D****T24**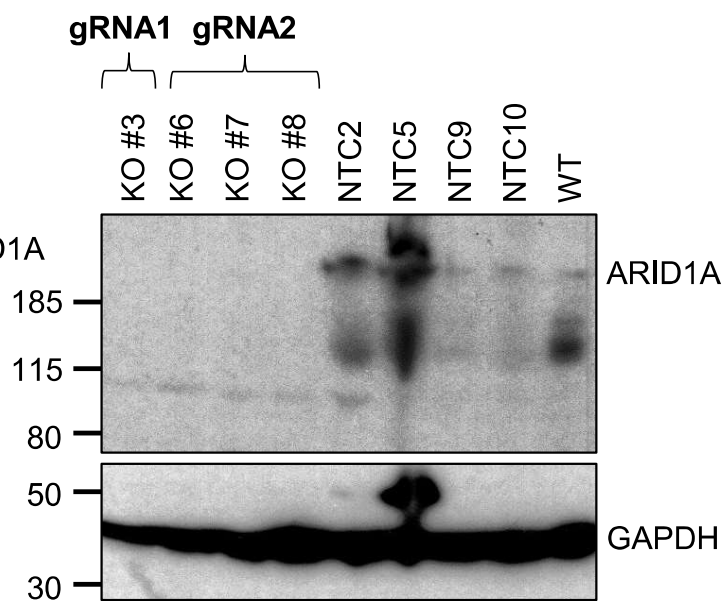

Supplement: Supplementary file 1 — Fig. S1. Western blots of all used models in this study showing ARID1A deficiency or proficiency, respectively. (A) HBLAK treated with or without siRNA against ARID1A. (B) CRISPR/Cas9‐mediated KO of ARID1A in UROtsa, KO clones are marked in red. (C) ARID1A re‐expression in JMSU‐1. (D) CRISPR/Cas9‐mediated KO of ARID1A in T24. [file MOL2-19-3784-s014.pdf]

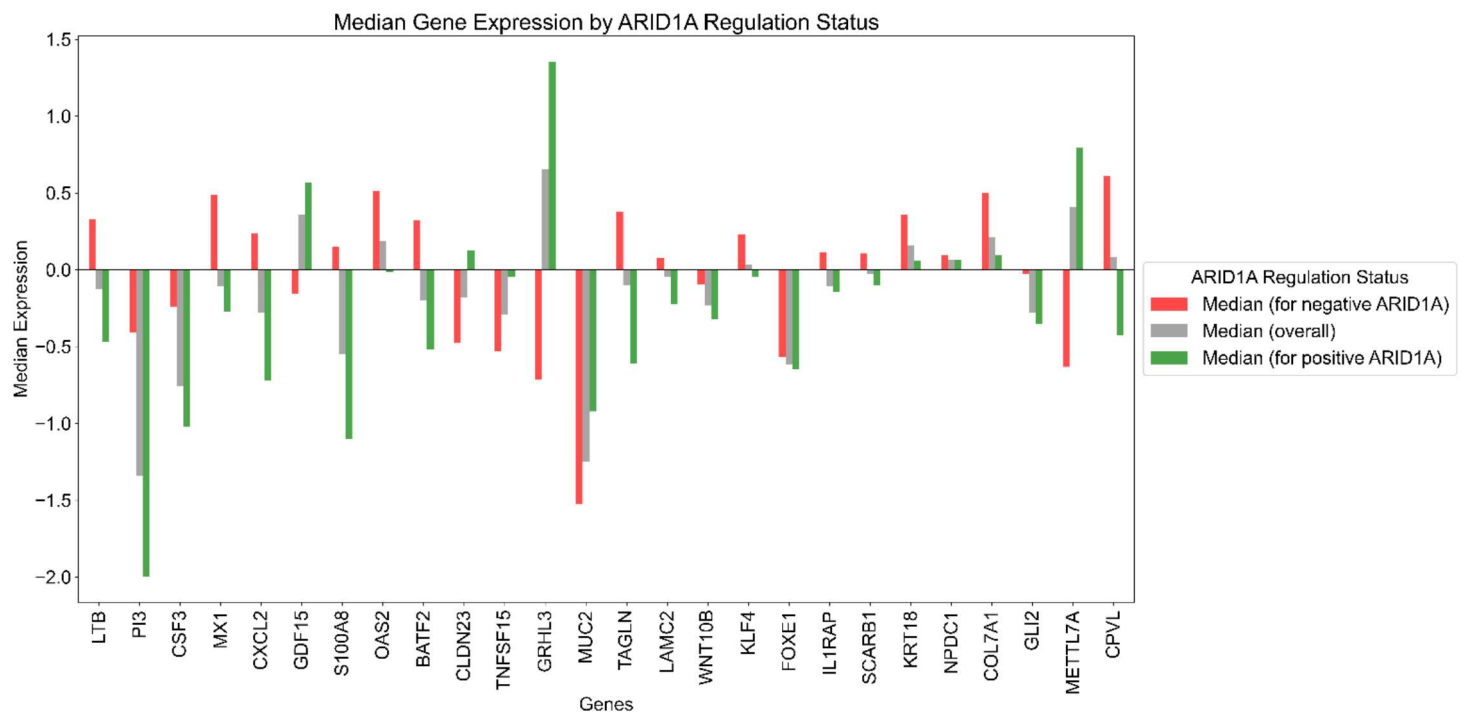

Supplement: Supplementary file 3 — Fig. S3. Translational regulation of genes found commonly regulated in UROtsa (ARID1A KO vs WT) and HBLAK (ARID1A KD vs WT) in TCGA 2017 cohort. Not all 47 significantly impacted genes were measured in the TCGA cohort. The median expression is shown for the median of negatively regulated ARID1A, positively regulated ARID1A, and overall regulation. [file MOL2-19-3784-s002.pdf]

**A**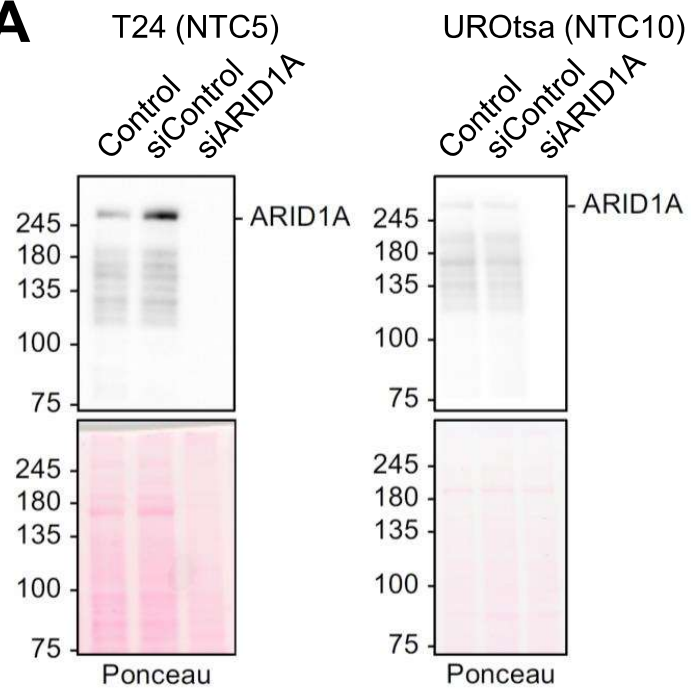**B**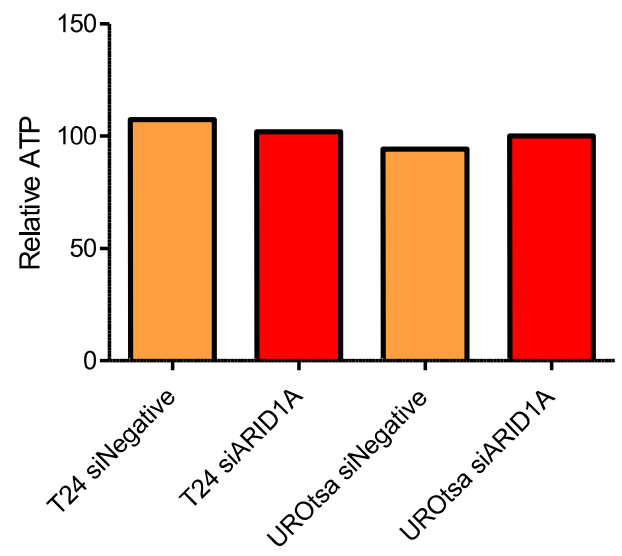

Supplement: Supplementary file 4 — Fig. S4. The effect of siARID1A KD on UROtsa and T24 cells. (A) Western blot and Ponceau staining of an siRNA KD in UROtsa and T24 cells. B: Bar chart results of a CellTiterGlo assay of UROtsa and T24 treated with siRNA against ARID1A. Each clone was transfected twice and seeded in triplicates for the CellTiterGlo measurements. Measurements for 48–72 h were averaged and shown. [file MOL2-19-3784-s011.pdf]

A

## Promoters

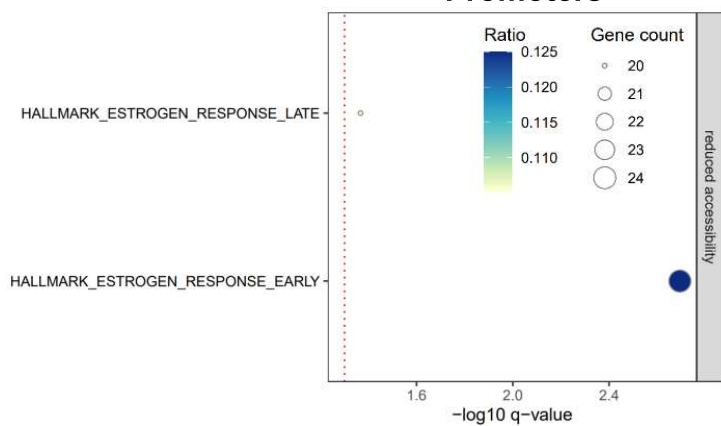

B

## Exon

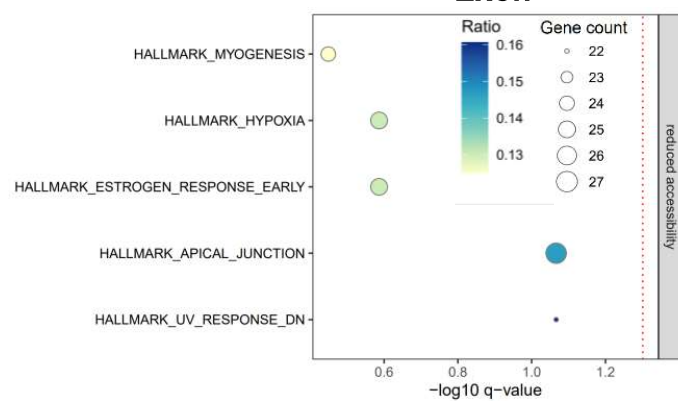

C

## Exon &amp; intron

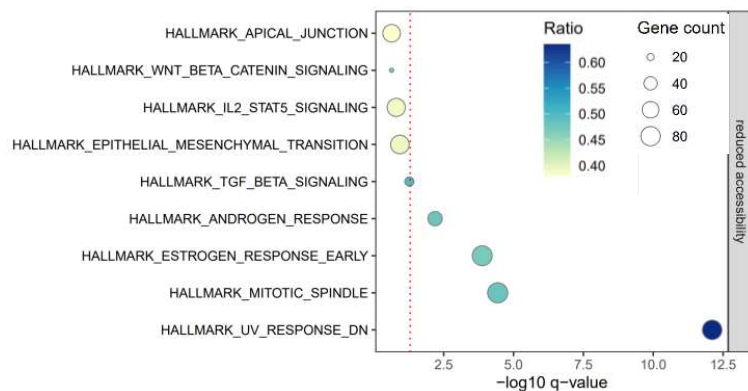

D

## Exon, intron &amp; 3'UTR

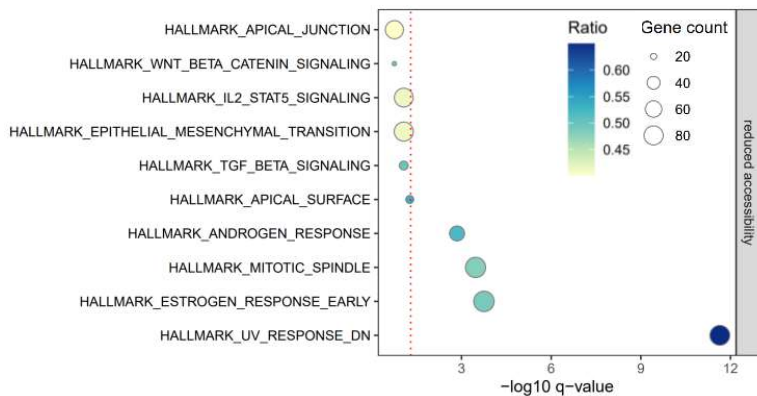

Supplement: Supplementary file 5 — Fig. S5. Hallmarks GSEA of ARID1A KO vs WT UROtsa from an Omni‐ATAC‐Seq of (A) promoters, (B) exons, (C) exons and introns, (D) exons, introns, and 3′UTR. [file MOL2-19-3784-s007.pdf]
