# Supplementary material for: Multidimensional OMICs reveal ARID1A orchestrated control of DNA damage, splicing, and cell cycle in normal‐like and malignant urothelial cells
Source: Mol Oncol. 2025 Apr 1;19(12):3784–805. doi: 10.1002/1878-0261.70019 (PMC12688176; doi:10.1002/1878-0261.70019)
Supplement: Supplementary file 2 — Fig. S2. Sanger Sequencing results of generated T24 and UROtsa ARID1A KO. Each panel shows the respective clone, wildtype sequence and sequence of the KO. (A) T24 ARID1A KO gRNA1 KO#3. (B) T24 ARID1A KO gRNA2 KO#6. (C) T24 ARID1A KO gRNA2 KO#7. (D) T24 ARID1A KO gRNA2 KO#8. (E) UROtsa ARID1A KO gRNA2 KO#14; (F) UROtsa ARID1A KO gRNA3 KO#13; (G) UROtsa ARID1A KO gRNA3 KO#29; (H) UROtsa ARID1A KO gRNA3 KO#31. [file MOL2-19-3784-s004.pdf]

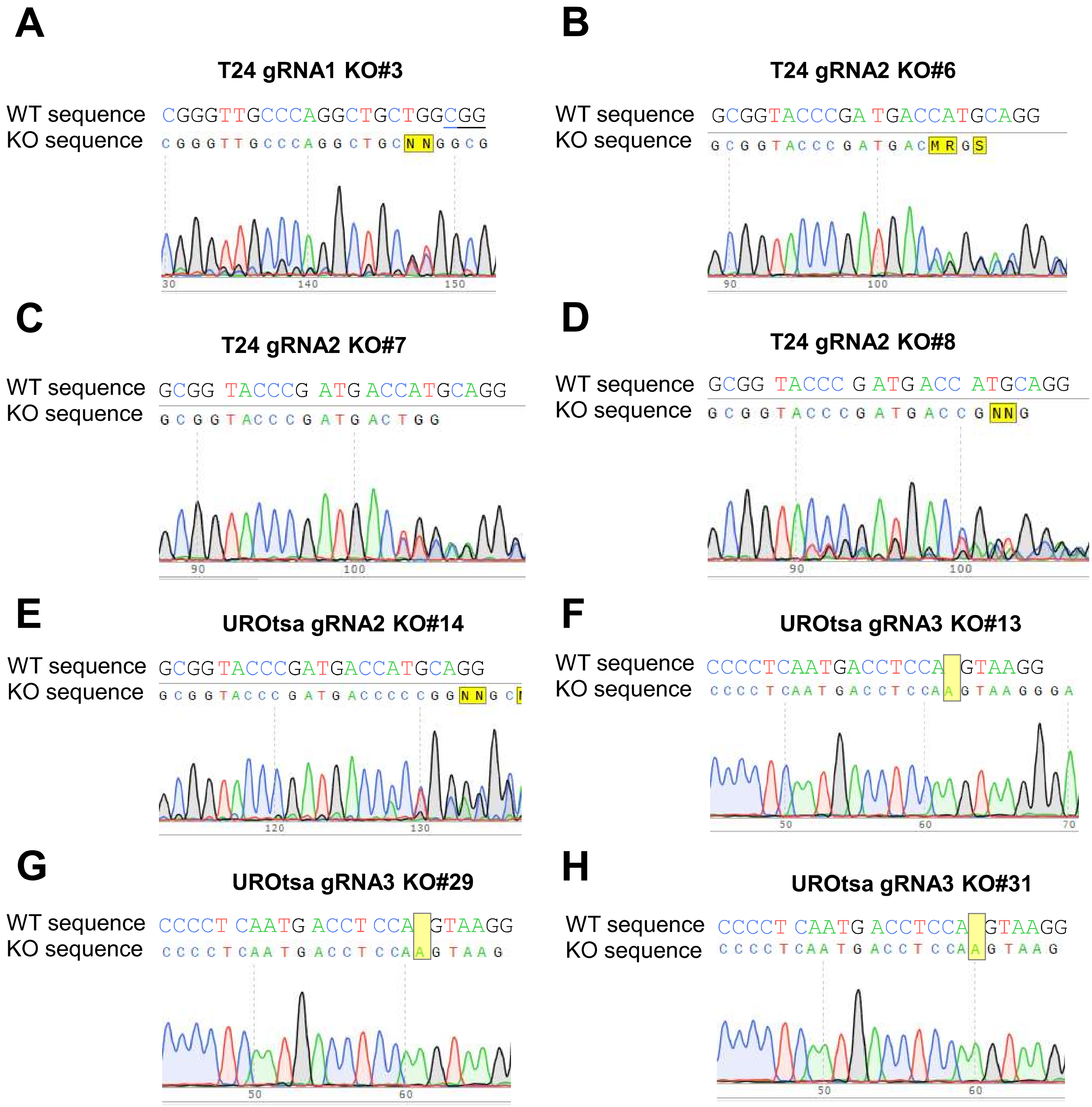
A
T24 gRNA1 KO#3
WT sequence CGGGTTGCCCAGGCTGCTGGCGG
KO sequence CGGGTTGCCCAGGCTGCNNGGCG
30 140 150
B
T24 gRNA2 KO#6
WT sequence GCGGTACCCGATGACCATGCAGG
KO sequence GCGGTACCCGATGACMRGS
90 100
C
T24 gRNA2 KO#7
WT sequence GCGG TACCCG ATG ACCATGCAGG
KO sequence GCGGTACCCGATGACTGG
90 100
D
T24 gRNA2 KO#8
WT sequence GCGG TACCC G ATG ACC ATGCAGG
KO sequence GCGGTACCCGATGACCGNNG
90 100
E
UROtsa gRNA2 KO#14
WT sequence GCGGTACCCGATGACCATGCAGG
KO sequence GCGGTACCCGATGACCCCGGNNGC
120 130
F
UROtsa gRNA3 KO#13
WT sequence CCCCTCAATGACCTCCA GTAAGG
KO sequence CCCCTCAATGACCTCCAAGTAAGGGA
50 60 70
G
UROtsa gRNA3 KO#29
WT sequence CCCCT CAATG ACCT CCA GTAAGG
KO sequence CCCCTCAATGACCTCCAAGTAAG
50 60
H
UROtsa gRNA3 KO#31
WT sequence CCCCT CAATG ACCTCCA GTAAGG
KO sequence CCCCTCAATGACCTCCAAGTAAG
50 60
