# Supplementary material for: Multidimensional OMICs reveal ARID1A orchestrated control of DNA damage, splicing, and cell cycle in normal‐like and malignant urothelial cells
Source: Mol Oncol. 2025 Apr 1;19(12):3784–805. doi: 10.1002/1878-0261.70019 (PMC12688176; doi:10.1002/1878-0261.70019)
Supplement: Supplementary file 14 — Table S1. Oligos used in this study. Guide RNA (gRNA) sequences are underlined. [file MOL2-19-3784-s012.pdf]

Table 1: Oligos used in this study. Guide RNA (gRNA) sequences are underlined.

Oligo	Sequence (5‘ to 3‘)	Sequence origin	Annealing temperature
siRNA ARID1A #4	CAGAGTTTACTCTGTACGAAT	Qiagen	
siRNA ARID1A #6	CTCGGTATCACCGTTGATGAA	Qiagen	
siRNA ARID1A #7	GGAGCUAUCUCAAGAUUCA	Qiagen	
Primer JMJD1C forward	GGTAAGCGGTTCCTGTGTGT	This paper	60°C
Primer JMJD1C reverse	TTTGGCCCAGATTAAGTGGT	This paper	60°C
Primer DHX29 forward	AAAATTGGCTTGCATTGTGG	This paper	60°C
Primer DHX29 reverse	ACGTTCTCGGTGCTGAACTT	This paper	60°C
gRNA1 ARID1A forward	CACCGCGGGTTGCCCAGGCTGCTGG	Bitler et al 2017	
gRNA1 ARID1A reverse	AAACCCAGCAGCCTGGGCAACCCGC	Bitler et al 2017	
gRNA1 sequencing primer forward	AGACAGCGGGGATCATGG	This paper	57°C
gRNA1 sequencing primer reverse	CGTCCTGCAGCTCCTTTC	This paper	57°C
gRNA1 off-target primer 1 forward	TTCAGAAGTCCCAAGCAGCC	This paper	57°C
gRNA1 off-target primer 1 reverse	AGGGTAGAAGATCCTGTGGCT	This paper	57°C
gRNA1 off-target primer 2 forward	TACTTCCCAGAACCGGTGAC	This paper	57°C
gRNA1 off-target primer 2 reverse	GTGTTGCTGGGCTTGTGATT	This paper	57°C
gRNA2 ARID1A forward	CACCGGCGGTACCCGATGACCATGC	Sanjana et al 2014 (GeCKov2)	
gRNA2 ARID1A reverse	AAACGCATGGTCATCGGGTACCGCC	Sanjana et al 2014 (GeCKov2)	
gRNA2 sequencing primer forward	CCAATGGATCAGATGGGCAAG	This paper	55°C
gRNA2 sequencing primer reverse	TTTGGACAGCAACAAGGGTC	This paper	55°C
gRNA2 off-target primer forward	TCCAAGTTCTTCCTCAGCCAC	This paper	56°C
gRNA2 off-target primer reverse	CATCCAGACCAGCTCCACC	This paper	56°C
gRNA3 ARID1A forward	CACCGCCCCTCAATGACCTCCAGTA	Sanjana et al 2014 (GeCKov2)	
gRNA3 ARID1A reverse	AAACTACTGGAGGTCATTGAGGGGC	Sanjana et al 2014 (GeCKov2)	
gRNA3 sequencing primer forward	GGCCATCACAGCTTTTGTTT	This paper	55°C
gRNA3 sequencing primer reverse	TTTCCTCTCTGCCCCTATCA	This paper	55°C
gRNA3 off-target primer 1 forward	ATAGGACTGCTGGAGGACC	This paper	57°C
gRNA3 off-target primer 1 reverse	CTGAGCTGCCACCTCCTATC	This paper	57°C
gRNA3 off-target primer 2 forward	TGCCATAGAAGAGGATGCTGT	This paper	57°C
gRNA3 off-target primer 2 reverse	TTCAATGGGGATGCAGACCT	This paper	57°C
gNTC off-target primer forward	CCTTGTCCTTCAGATGCAGC	This paper	57°C
gNTC off-target primer reverse	GCGTTGAAGCTGCAGAAACT	This paper	57°C
